# Splenic B-1a Cells Expressing CD138 Spontaneously Secrete Large Amounts of Immunoglobulin in Naïve Mice

**DOI:** 10.3389/fimmu.2014.00129

**Published:** 2014-03-28

**Authors:** Nichol E. Holodick, Teresa Vizconde, Thomas L. Rothstein

**Affiliations:** ^1^Center for Oncology and Cell Biology, The Feinstein Institute for Medical Research, Manhasset, NY, USA; ^2^Departments of Medicine and Molecular Medicine, Hofstra North Shore-LIJ School of Medicine, Manhasset, NY, USA

**Keywords:** B lymphocytes, antibody, B-1 cells, IgM

## Abstract

B-1a cells constitutively secrete natural antibody that provides immediate protection against microbial pathogens and functions homeostatically to speed removal of apoptotic cell debris. Although B-1a cells are especially prominent in the peritoneal and pleural cavities, some B-1a cells reside in the spleen. A small subset of splenic B-1a cells in naïve, unimmunized mice express CD138, a recognized plasma cell antigen, whereas the bulk of splenic B-1a cells are CD138 negative. Splenic B-1a cells *in toto* have been shown to generate much more antibody per cell than peritoneal B-1a cells; however, specific functional information regarding CD138^+^ splenic B-1a cells has been lacking. Here, we find a higher proportion of CD138^+^ splenic B-1a cells spontaneously secrete more IgM as compared to CD138^−^ B-1a cells. Moreover, IgM secreted by CD138^+^ splenic B-1a cells is skewed with respect to N-region addition, and some aspects of V_H_ and J_H_ utilization, as compared to CD138^−^ splenic B-1a cells and peritoneal B-1a cells. The small population of CD138^+^ splenic B-1a cells is likely responsible for a substantial portion of natural IgM and differs from IgM produced by other B-1a cell subsets.

## Introduction

Murine B-1a cells represent a unique lymphocyte lineage distinguished by specific ontologic, phenotypic, and functional characteristics (1, 2). Although the human equivalent of mouse B-1a cells has been described recently (3, 4), most knowledge about B-1a cells has been generated from studies in mice. It has been shown that B-1a cells spontaneously and constitutively generate “natural” immunoglobulin (Ig), which constitutes the vast majority of resting serum IgM and about half of resting IgA (5). The natural Ig produced by B-1a cells differs from Ig produced by B-2 cells. B-1a cell Ig contains minimal N-region addition and little somatic hypermutation (6–9). As a result, B-1a cell Ig tends to be “germline-like”; that is, to accurately reflect germline sequences without the intervention of randomly added nucleotides or mutated residues. Because B-1a cell Ig reflects germline coding, the B-1a cell repertoire is to a large extent inherited. Therefore, those sequences that enhance an organism’s survival to reproductive age are likely to be retained.

B-1a cell Ig is both polyreactive and autoreactive. Polyreactive natural Ig is always present and acts as an initial shield against many common infectious agents, particularly during the lag period that precedes the development of adaptive, high affinity antigen-specific antibody produced by germinal center B-2 cells (10–15). Autoreactive natural Ig has been implicated in the disposition of irreversibly damaged cells and noxious molecular debris and in so doing maintains homeostasis and prevents untoward inflammation (16).

B-1a cells are preferentially located at serosal surfaces, most prominently in the peritoneal cavity. However, B-1a cells are also located in the spleen, and splenic B-1a cells differ from peritoneal B-1a cells in a number of characteristics, including the intensity of Ig secretion (17–19). Through the unique property of self-renewal (20), mature B-1a cells can give rise to their own progeny in place, which suggests differentiation and migration in adult animals is minimal. Therefore, B-1a cells were considered for a time to represent static, Ig generating lymphocytes. However, recent reports suggest a richer life experience. Peritoneal B-1a cells may respond to specific and/or non-specific stimulation, migrate to the spleen, and may then return to the peritoneal cavity as memory B-1a cells (21–23). Further, a subset of splenic B-1a cells expresses CD138 (24) suggesting that appropriately stimulated B-1a cells may take a different path and differentiate in a plasma cell direction. CD138^+^ B-1a cells differ from CD138^+^ plasma cells in retaining B-1a-specific surface antigen expression. Identification of splenic B-1a cells that express CD138 raises the question of whether CD138^+^ B-1a cells differ from CD138^−^ B-1a cells, or whether these splenic B-1a cell populations behave similarly, especially with respect to the amount of Ig secretion.

## Materials and Methods

### Mice

Male BALB/c-ByJ mice of 6–8 weeks age were obtained from the Jackson Laboratory. Mice were cared for and handled in accordance with National Institutes of Health and institutional guidelines.

### Cell purification and flow cytometry

Splenocytes were obtained from 8 to 14-week-old BALB/c-ByJ male mice and stained with fluorescence-labeled antibodies to B220, CD5, CD23, and CD138. Splenic B cell populations were sort-purified (BD Biosciences Influx) as follows: splenic B-2 cells, B220^hi^CD5^−^CD23^hi^CD138^−^; splenic CD138^+^ B-1a cells, B220^lo^CD5^lo^CD23^−^CD138^+^; splenic CD138^−^ B-1a cells, B220^lo^CD5^lo^CD23^−^CD138^−^. Post-sort analysis of the splenic B-1a and B-2 cell populations showed each to be ≥98% pure. The following rat anti-mouse antibodies were obtained from BD Biosciences: FITC-conjugated B220 (clone RA3–6B2), PE-Cy5-conjugated CD5 (clone 53–7.3), PE-conjugated CD138 (clone 281–2). The anti-mouse Pacific Blue-conjugated CD23 antibody (clone B3B4) was obtained from BioLegend.

### ELISPOT assay

ELISPOT assay was carried out as previously described (25). In brief, sort-purified, naive B cells were distributed onto MultiScreen*-IP Plates (Millipore) pre-coated with goat anti-mouse Ig (H + L) and then incubated in RPMI 1640 containing 10% heat-inactivated fetal bovine serum, 2 mM l-glutamine, 50 μM 2-mercaptoethanol, 100 U/ml penicillin, and 100 μg/ml streptomycin for 4 h at 37°C and 5% CO_2_. Plates were treated with alkaline phosphatase-conjugated goat anti-mouse IgM (Southern Biotechnology Associates) and developed with 5-bromo-4-chloro-3-indolyl phosphate/p-NBT chloride substrate (KPL). IgM-secreting B cells were enumerated using Phoretix Expression software (Non-Linear Dynamics).

### Single cell sequencing and analysis

Splenic CD138^+^ B-1a and CD138^−^ B-1a cells were sorted onto 48-well AmpliGrid slides (Advalytix). Reverse transcription and PCR (Qiagen OneStep RT-PCR) were carried out as described previously (8). The products were purified and then sequenced (Genewiz) using the MsVHE primer. Sequences were then analyzed using an online sequence analysis tool for VDJ sequences (IMGT, the international ImMunoGeneTics information system). Each of the sequences analyzed and reported in this manuscript, from each population, is characterized by a unique V, D, and J segment along with a unique CDR3. Sequences with identical V, D, and J segments as well as identical CDR3 regions were eliminated from consideration according to the criteria of Kantor et al. (26). CDR3 hydrophobicity was calculated using the online calculator GRAVY (http://www.gravy-calculator.de/index.php?page=file). Sequences are provided as Data Sheet in Supplementary Material.

## Results

**Figure 1:**
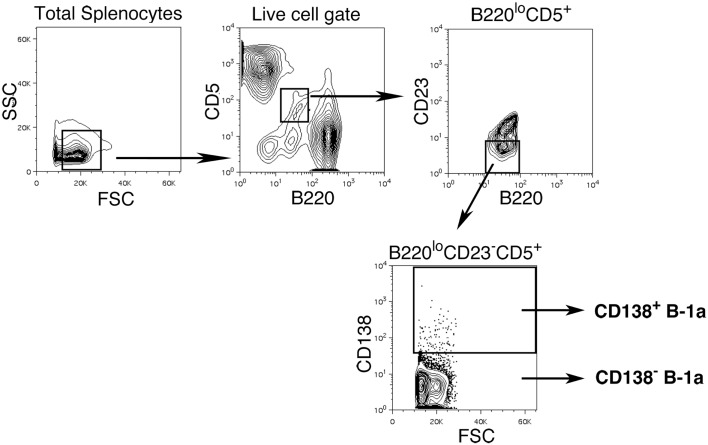
**Gating strategy for CD138^+^ and CD138^−^ splenic B-1a cells in naïve non-immune mice**. Single cell suspensions of RBC lysed spleens from BALB/c-ByJ mice were prepared and stained with B220-FITC, CD5-PE-Cy5, CD23-Pacific Blue, and CD138-PE. Splenic CD138^+^ and CD138^−^ B-1a cells were gated as shown and sorted.

**Figure 2:**
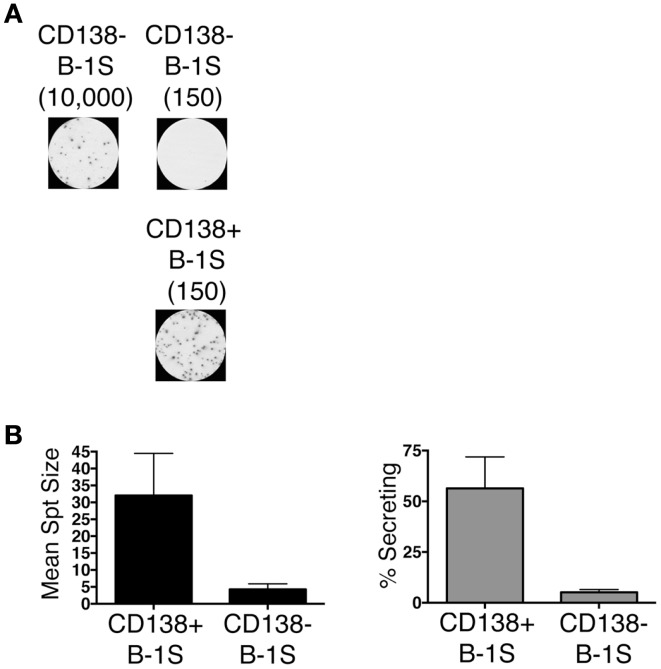
**CD138^+^ and CD138^−^ splenic B-1a cell IgM secretion in naïve non-immune mice**. Sorted splenic CD138^+^ and CD138^−^ B-1a cells were evaluated for IgM secretion by ELISPOT. Cells were placed on the ELISPOT membrane for 4 h. **(A)** Representative experiment showing 10,000 or 150 CD138^−^ cells total placed per well as compared to 150 CD138^+^ cells total per well. **(B)** The spots were enumerated to determine mean spot area (black) and the percent of cells secreting IgM (gray). Results shown represent mean values of three independent experiments with lines indicating standard errors of the means.

We sort purified splenic B-1a cells to separate CD138^+^ and CD138^−^ populations using a variation on the gating strategy reported by Herzenberg and colleagues (24). The selected splenic populations were gated as B220^lo^CD5^lo^CD23^−^CD138^+^ (CD138^+^ B-1a cells) and B220^lo^CD5^lo^CD23^−^CD138^−^(CD138^−^ B-1a cells), as depicted in Figure 1. Far fewer CD138^+^ B-1a cells were recovered than CD138^−^ B-1a cells with the former amounting to less than 1/100 the number of the latter. We questioned whether CD138 expression marks splenic B-1a cells that vigorously secrete Ig, as it does B-2 plasma cells. To address this, we tested IgM secretion of sorted splenic B-1a cells from naïve, unimmunized BALB/c-ByJ mice by ELISPOT assay. We found both CD138^+^ and CD138^−^ B-1a cells secreted IgM spontaneously, without stimulation, over a 4 h period. However, the frequency of IgM-secreting B-1a cells was significantly higher for CD138^+^ B-1a cells in comparison to CD138^−^ B-1a cells, as illustrated in Figure 2A and enumerated in Figure 2B. More than half of CD138^+^ splenic B-1a cells (56%) spontaneously secreted IgM whereas the fraction of CD138^−^ splenic B-1a cells that secreted IgM (5%) was much lower. Beyond frequency, the amount of IgM secreted by CD138^+^ splenic B-1a cells was significantly greater than that of CD138^−^ splenic B-1a cells, as judged by relative mean spot area (Figure 2B). CD138^+^ B-1a cells generated ELISPOTS that were more than six times as large as the ELISPOTS produced by CD138^−^ B-1a cells. Still, both CD138^+^ and CD138^−^ splenic B-1a cells secreted more IgM per cell than peritoneal B-1a cells (18).

**Figure 3:**
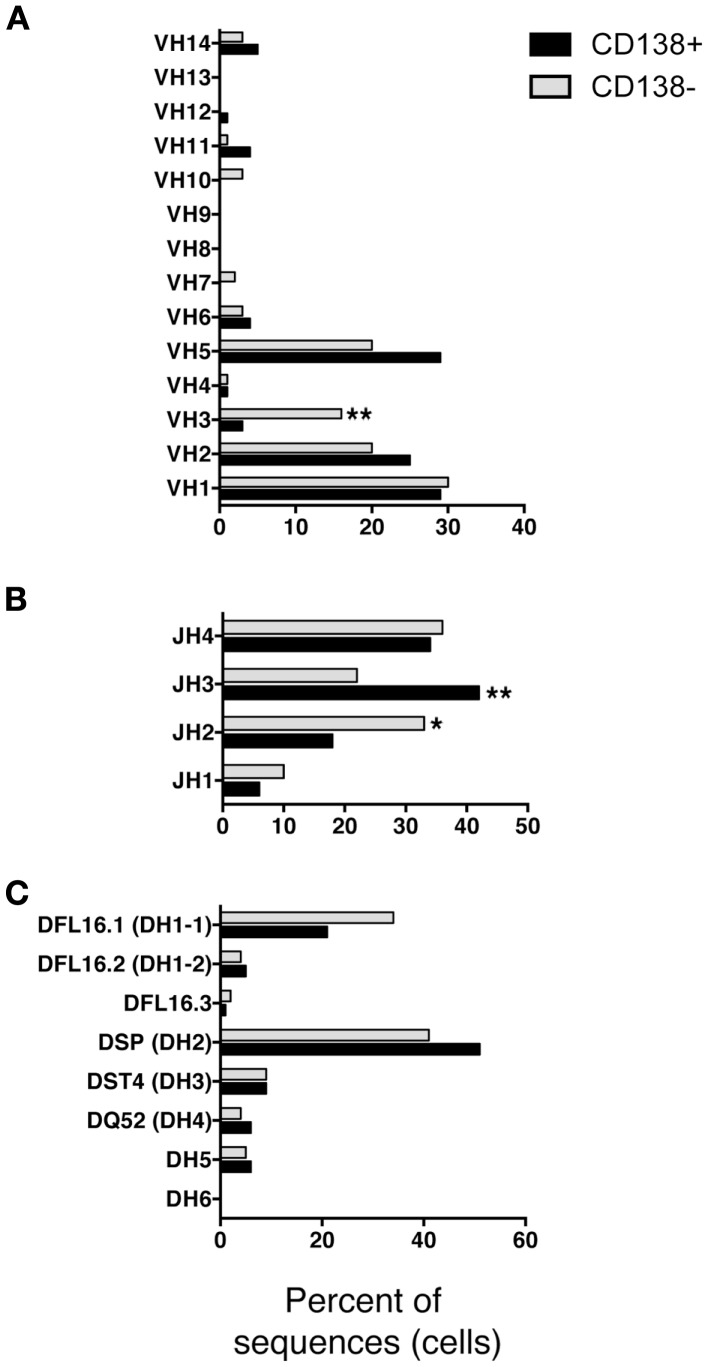
**VH, DH, and JH analysis of CD138^+^ and CD138^−^ splenic B-1a cells**. Immunoglobulins were amplified by PCR from single sorted splenic CD138^+^ (*n* = 77) and CD138^−^ (*n* = 92) B-1a cells and evaluated for **(A)** V, **(B)** J, and **(C)** D segment heavy chain usage. The percent of cells (sequences) expressing each segment is displayed. Chi square and Fisher’s exact tests were used to determine significance.

We questioned whether IgM produced by CD138^+^ B-1a cells represents a selected repertoire in comparison to CD138^−^ B-1a cells. To address this, we sorted single cell CD138^+^ and CD138^−^ splenic B-1a cells and analyzed individual antibodies by PCR amplification and sequencing. We found overall similarity in V_H_–D_H_–J_H_ usage with several significant differences. Among V_H_ gene segments, V_H_3 was expressed significantly less frequently by CD138^+^ splenic B-1a cells (3%; *n* = 77; Figure 3A) as compared to CD138^−^ splenic B-1a cells (16%; *n* = 92; Figure 3A) (*p* = 0.003). CD138^+^ splenic B-1a cells also expressed V_H_3 less frequently than peritoneal B-1a cells [13%; *n* = 56; Ref. (8)] (*p* = 0.02). In contrast, V_H_5 was expressed more frequently by CD138^+^ splenic B-1a cells (29%) as compared to CD138^−^ splenic B-1a cells (20%), although this difference did not reach the level of significance. Among D_H_ gene segments, DFL16.1 was expressed less frequently, and DSP was expressed more frequently, by CD138^+^ splenic B-1a cells as compared to CD138^−^ splenic B-1a cells although these differences were not significant (Figure 3C). Among J_H_ gene segments, J_H_3 was expressed significantly more frequently (42%; Figure 3B) (*p* = 0.005) and J_H_2 was expressed significantly less frequently (18%; Figure 3B) (*p* = 0.03), by CD138^+^ splenic B-1a cells as compared to CD138^−^ splenic B-1a cells (22 and 33%, respectively). The latter utilization of J_H_3 and J_H_2 by CD138^−^ splenic B-1a cells approximated that of peritoneal B-1a cells [18 and 27%, respectively, Ref. (8)]. Thus, distinctive V and J gene segment usage separated CD138^+^ splenic B-1a cells from CD138^−^ splenic B-1a cells and peritoneal B-1a cells.

**Figure 4:**
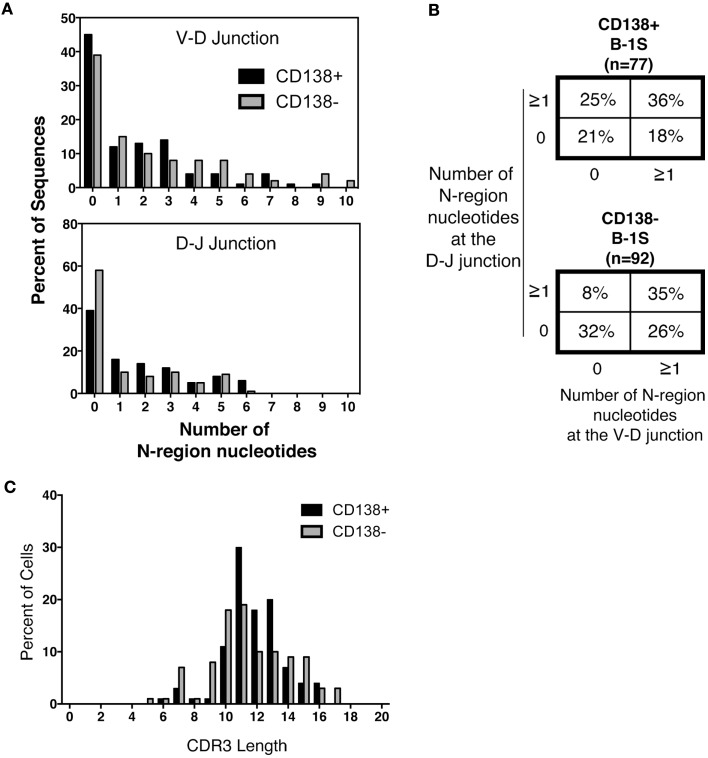
**N-region addition and CDR3 length analysis of CD138^+^ and CD138^−^ splenic B-1a cells**. Immunoglobulins were amplified by PCR from single sorted splenic CD138^+^ (*n* = 77) and CD138^−^ (*n* = 92) B-1a cells and evaluated for N-region additions and CDR3 lengths. **(A)** The number of N-additions at each junction is shown. The graphs display the percent of sequences with 0–10 N-additions at each junction for CD138^+^ (black bars) and CD138^−^ (gray bars) splenic B-1a cells. **(B)** N-addition analysis of the V–D and D–J junctions together. **(C)** CDR3 length analysis of immunoglobulin sequences from CD138^+^ (black bars) and CD138^−^ (gray bars) splenic B-1a cells.

Much attention has focused on N-region addition in B-1a cell Ig, which is typically severely limited in comparison to B-2 cell Ig. We questioned whether CD138^+^ B-1a cell Ig represents a selected subset of all B-1a cell Ig. To address this, we analyzed N-addition at the D–J and V–D junctions and determined CDR3 length. Analyzing the junctions separately, we found the average length of N-additions at the D–J junction of CD138^+^ splenic B-1a cell antibodies was larger than that of CD138^−^ splenic B-1a cells (*p* = 0.03 by Mann–Whitney *U*) (Table 1). This bespeaks increased diversity among antibodies expressed by CD138^+^ B-1a cells as compared to CD138^−^ B-1a cells. However, there were no significant differences between CD138^+^ and CD138^−^ splenic B-1 cell antibodies at V–D junction in terms of mean N-addition length. Regardless, overall N-addition in CD138^+^ splenic B-1a cell Ig was significantly different (*p* = 0.003 by Chi square analysis) from that of CD138^−^ splenic B-1a cell Ig (Figure 4B), emphasizing that CD138 expression divides splenic B-1a cells into distinct populations.

**Table 1:** **Mean N-region addition and CDR3 lengths (±SD)**.

	V–D	D–J	CDR3 length
CD138^+^ splenic B-1a	1.7 (±2.2)	1.8 (±1.9)	11.8 (±1.9)
CD138^−^splenic B-1a	2.3 (±2.8)	1.3 (±1.8)	11.6 (±2.6)

In previous work, we analyzed N-addition frequency of Ig sequences amplified from peritoneal B-1a cells and from splenic B-2 cells (8). Antibodies expressed by both CD138^+^ and CD138^−^ splenic B-1a cells contained significantly more N-additions (Figure 4A) in comparison to antibodies expressed by B-1a cells in the peritoneal cavity, by Chi square analysis (Figure 4B and Ref. (8); *p* < 0.0001 and *p* = 0.02, respectively). Conversely, antibodies expressed by both CD138^+^ and CD138^−^ splenic B-1a cells contained significantly fewer N-additions in comparison to antibodies expressed by splenic B-2 cells, by Chi square analysis (Figure 4B and Ref. (8); *p* = 0.0003 and *p* < 0.0001, respectively). Thus, whereas Ig sequences from CD138^+^ and CD138^−^ splenic B-1a cells differ in N-addition, both populations of splenic B-1a cells differ from peritoneal B-1a cells and splenic B-2 cells, whose Ig contains less and more N-addition, respectively.

We further examined CDR3 length and found that despite differences in N-addition between CD138^+^ and CD138^−^ splenic B-1a cells, average Ig CDR3 lengths were not significantly different for the two populations (Table 1; Figure 4C). Moreover, average Ig CDR3 hydrophobicity indices were similar for CD138^+^ and CD138^−^ splenic B-1a cell antibodies (−0.63 ± 0.065 and −0.69 ± 0.064 SEM, respectively). Thus, the small CD138^+^ splenic B-1a cell population expresses Ig that varies in Ig gene segment usage and is more diverse on account of increased N-addition, as compared to the dominant CD138^−^ splenic B-1a cell population, but differs little in CDR3 length and CDR3 hydrophobicity.

## Discussion

Our results further characterize CD138-bearing B-1a cells, first identified by Yang et al. (24), which appear as a very small B cell population in the spleen. A large fraction of this small population secretes IgM, and an increased amount of IgM is secreted, in comparison with CD138^−^ splenic B-1a cells and peritoneal B-1a cells. Thus, although their numbers may be small, it is likely that CD138^+^ splenic B-1a cells make a substantial contribution to the circulating pool of natural antibodies. Therefore, the nature of the CD138^+^ antibody repertoire is of interest in understanding the protective capacity of natural IgM.

The repertoire of these potently secreting CD138^+^ splenic B-1a cells is somewhat skewed away from V_H_3 and J_H_2, and toward J_H_3. This would seem to parallel the situation with the peritoneal B-1a cell repertoire, in which overall usage of V_H_ gene segments is similar to that of B-2 cells except for the key difference of increased V_H_11 and V_H_12 expression, the two V_H_ families responsible for PtC binding (27–29). In other words, repertoire skewing can show up in a limited way, which may be the case here with the preferential use of a very small number of V_H_ and J_H_ gene segments by CD138^+^ splenic B-1a cells.

Prominent among variably expressed V_H_ gene segments is V_H_3, which was found to be increased in NZM2410 anti-nuclear antibodies as compared to antibodies that did not bind nuclear components (30). This might suggest that CD138^+^ splenic B-1a cells predominantly generate anti-microbial as opposed to self-reactive antibodies; however, in NZM2410 mice V_H_5 is also increased in anti-nuclear antibodies (30) and among splenic B-1a cells V_H_5 is utilized more frequently by CD138^+^ B-1a cells as compared to CD138^−^ B-1a cells, although this difference did not reach statistical significance. Thus, there is no clear evidence that CD138^+^ B-1a cell Ig skews more toward or away from autoreactivity.

Both CD138^+^ and CD138^−^ splenic B-1a cell Ig sequences contained more N-addition than peritoneal B-1a cells’ Ig sequences (and less N-addition than splenic B-2 cell Ig sequences), suggesting that the splenic B-1a pool differs from B-1a cells located elsewhere as well as from B-2 cells that share the splenic environment. Moreover, among splenic B-1a cell Ig, CD138^+^ B-1a sequences contained more N-addition than CD138^−^ sequences. It has been shown the natural IgM produced by B-1a cells is essential for early protection against bacterial and viral infections and that N-addition plays a substantial role in determining antibody diversity and effectiveness (10, 11, 13, 15). For example, the prototypical B-1a anti-phosphorylcholine (PC) antibody, T15, represents a germline sequence and has no N-addition (31). T15 has been shown to be protective against *Streptococcus pneumoniae* infection (31). The relationship between N-addition and antibody function is illustrated by the finding that after vaccination with heat killed pneumococci, mice that overexpress TdT generated an anti-PC response, but the anti-PC antibodies in this situation were not protective against *S. pneumoniae* infection (32). These findings highlight the importance of N-addition, which varies among antibodies spontaneously secreted by CD138^+^ splenic B-1a cells, CD138^−^ B-1a splenic B-1a cells, and peritoneal B-1a cells, in determining protection by natural antibody.

Circulating natural antibody is primarily generated by splenic B-1a cells, which differ in many characteristics from peritoneal B-1a cells (17–19). Among splenic B-1a cells, CD138^+^ B-1a cells differ from CD138^−^ B-1a cells in the frequency of secreting cells, the amount of antibody secreted, and the repertoire of antibody expressed. The combination of skewing with respect to V_H_ and J_H_ gene segments, and degree of N-region addition, suggests that the CD138^+^ B-1a cell pool does not result from randomly triggered differentiation events applied to all splenic B-1a cells or all peritoneal B-1a cells, but rather results from a selective process whose origin remains unclear.

This raises the question of how the distinct splenic B-1a populations come about, and whether this represents selection from a pre-existing population or contribution from a new or different source. Previous work suggests several potential mechanisms. Peritoneal B-1a cells may migrate to the spleen following antigen-specific (or non-specific) activation (21–23, 33, 34). Herzenberg and colleagues have shown that these B-1a cells may become antibody secreting cells and/or return to the peritoneal cavity as memory B cells (21–23, 33, 34). In addition, we and others have suggested that the pool of B-1a cells changes with age, as fetal liver-derived B-1a cells are slowly replaced by bone marrow-derived B-1a cells in the adult expressing antibody with increased levels of N-addition (8, 35, 36), and the latter could preferentially give rise to splenic B-1a cells. A further possibility relates to the report of B-1 progenitor cells in the spleen that might give rise to mature B-1a cells *in situ* (37, 38). In fact, a combination of these mechanisms may be at play, whereby the fetal liver B-1a pool in the peritoneal cavity is replaced by bone marrow-derived B-1a emigrants over time, which then become activated and migrate to the spleen in a selective fashion. It will be of interest to determine whether the N-addition and other characteristics of CD138^+^ B-1a cells change with advancing age. In sum, CD138^+^ splenic B-1a cells constitute a distinct B-1a cell population that appears to play a substantial role in generation of natural antibody.

## Supplementary Material

The Supplementary Material for this article can be found online at http://www.frontiersin.org/Journal/10.3389/fimmu.2014.00129/abstract.

## Conflict of Interest Statement

The authors declare that the research was conducted in the absence of any commercial or financial relationships that could be construed as a potential conflict of interest.
