# Supplementary material for: Splenic B-1a Cells Expressing CD138 Spontaneously Secrete Large Amounts of Immunoglobulin in Naïve Mice
Source: Front Immunol. 2014 Mar 28;5:129. doi: 10.3389/fimmu.2014.00129 (PMC3975111; doi:10.3389/fimmu.2014.00129)
Supplement: Supplementary file 1 [file Data_Sheet1.PDF]

CD138+ Splenic B-1a Cells

Input	V name	3'V-REGION	P	N1	P	D-REGION	P	N2	P	5'J-REGION	J name	D name
168-E10	Musmus IGHV1-12*01	tgtgc....				....ctatggtaac...				..tactatgctatggactactgg	Musmus IGHJ4*01	Musmus IGHD2-1*01
167-A10	Musmus IGHV11-2*02	tgtatgagata				..........cggtagtag....				...ctggtacttcgatgtctgg	Musmus IGHJ1*01	Musmus IGHD1-1*01
168-G09	Musmus IGHV11-2*02	tgtatgagata				.......tggtaa....				ctactggtacttcgatgtctgg	Musmus IGHJ1*01	Musmus IGHD2-1*01
167-A09	Musmus IGHV1-37*01	tgtggaaga	gg			.......tggttactac		t		cctggtttgcttactgg	Musmus IGHJ3*01	Musmus IGHD2-3*01
167-C08	Musmus IGHV1-4*01	tgtgcaa..				....ctggg..		g		...ggtttgcttactgg	Musmus IGHJ3*01	Musmus IGHD4-1*01
167-A03	Musmus IGHV14-3*02	tgtgc....	c			..cattactac......		ctc	gt	actactttgactacggg	Musmus IGHJ2*01	Musmus IGHD1-2*01
167-A04	Musmus IGHV14-4*02	tgtaatgca	t			........tcgggcgac		g	gt	actactttgactacggg	Musmus IGHJ2*01	Musmus IGHD3-1*01
167-D01	Musmus IGHV1-55*01	tgtgcaaga	cacg			...actatggttac...		tct		.....tttgcttactgg	Musmus IGHJ3*01	Musmus IGHD2-2*01
167-B08	Musmus IGHV2-3*01	tgtgccaa...	ctgg			.....tactacggctac		ggagc		.ttactatgctatggactactgg	Musmus IGHJ4*01	Musmus IGHD1-2*01
167-D10	Musmus IGHV2-3*01	tgtgcc.....	gaccaa			..aactggg..				...acgatgctattgactactgg	Musmus IGHJ4*01	Musmus IGHD4-1*01
167-B04	Musmus IGHV2-6-4*01	tgtgccagaa.	gtctctcg			..tactatggta.....				...actttgactactgg	Musmus IGHJ2*01	Musmus IGHD2-1*01
169-B08	Musmus IGHV2-6-4*01	tgtgccagaa.	gg			..tatgatggttactac		gat		..tgttttgtttactgg	Musmus IGHJ3*01	Musmus IGHD2-3*01
167-D04	Musmus IGHV2-6-5*01	tgtgccaaaca				.......ctacggtagtagct..				attactatgctatggactactgg	Musmus IGHJ4*01	Musmus IGHD1-1*01
167-C09	Musmus IGHV2-9-1*01	tgtgccagaga	ggggt			cctactataggtacg..		gt		.....tttgcttactgg	Musmus IGHJ3*01	Musmus IGHD2-14*01
168-F06	Musmus IGHV3-2*02	tgtgcaa..	tg			agagaggctgcc................		g		cctggtttgcttactgg	Musmus IGHJ3*01	Musmus IGHD5-8*01
169-C07	Musmus IGHV5-12-2*01	tgtgcaagaca	c			.....gatgg.......		acc	gt	actactttgactactgg	Musmus IGHJ2*01	Musmus IGHD2-3*01
167-A06	Musmus IGHV5-12-2*01	tgtgcaagaca				....ttactacggtagtagct..				actactttgactactgg	Musmus IGHJ2*01	Musmus IGHD1-1*01
167-A12	Musmus IGHV5-4*02	tgtgcaagaga	tc	agg		.........gttactatag.......		gcccc		attactatgctatggactactgg	Musmus IGHJ4*01	Musmus IGHD2-12*01
169-B02	Musmus IGHV5-6*01	tgtgcaagaca	t	aag		........ggtaact..		c		.ctactttgactactgg	Musmus IGHJ2*01	Musmus IGHD2-1*01
168-G03	Musmus IGHV5-9-3*01	tgtgcaag...	tac			....gtatggtaactac		gtttt		.ttactatgctatggactactgg	Musmus IGHJ4*01	Musmus IGHD2-10*02
167-C05	Musmus IGHV5-9-4*01	tgtgcaagg..				.....tactacggtagtag....				.ctactttgactactgg	Musmus IGHJ2*01	Musmus IGHD1-1*01
167-C03	Musmus IGHV6-6*02	tgtacca..				.......tgattacga.				cctggtttgcttactgg	Musmus IGHJ3*01	Musmus IGHD2-4*01
155-A01_Ms	Musmus IGHV1-37*01	tgtggaaga		ac		....ttactacggct..				actactttgactactgg	Musmus IGHJ2*01	Musmus IGHD1-2*01
156-E04_Ms	Musmus IGHV1-14*01	tgtgcaaga		gg		....gtatggtaa....				.ctggtttgcttactgg	Musmus IGHJ3*01	Musmus IGHD2-10*02
171-B07_Ms	Musmus IGHV1-14*01	tgtgcaaga		c		.........gtaac...				..tacgacgctatggactactgg	Musmus IGHJ4*01	Musmus IGHD2-1*01
171-D04_Ms	Musmus IGHV5-12-2*01	tgtgcaagaca		a		...actatgattacga.				cctggtttgcttactgg	Musmus IGHJ3*01	Musmus IGHD2-4*01
171-D06_Ms	Musmus IGHV1-4*01	tgtgcaaga		cgg		...........gggagta.....				...actatgctatggactactgg	Musmus IGHJ4*01	Musmus IGHD1-1*01
172-E03_Ms	Musmus IGHV2-9-1*01	tgtgccag...		cc		.......cttgg.....				........gcttactgg	Musmus IGHJ3*01	Musmus IGHD3-1*01
172-E10_Ms	Musmus IGHV1-18*01	tgtgcaag.		g		ggggtacctcggg....				cctggtttgcttactgg	Musmus IGHJ3*01	Musmus IGHD3-3*01
172-G07_Ms	Musmus IGHV1-26*01	tgtgcaaga		cc		..acagtacgcc................				..tactatgctatggactactgg	Musmus IGHJ4*01	Musmus IGHD5-8*01
172-H03_Ms	Musmus IGHV1-14*01	tgtgcaaga		aggaggggt		.....tatggta.....				actggtttgactactgg	Musmus IGHJ2*01	Musmus IGHD2-1*01
174-C08_Ms	Musmus IGHV1-14*01	tgtgcaag.		ggggggc		..tattactacggtagtag....				.ctactttgactactgg	Musmus IGHJ2*01	Musmus IGHD1-1*01
156-F06_Ms	Musmus IGHV1-5*01	tgcactag.				..........cggt.........				..tggtttgcttactgg	Musmus IGHJ3*01	Musmus IGHD1-1*01
171-B10_Ms	Musmus IGHV12-1-1*01	tgttccagggaaaac..				........tcggg....				........gctatggactactgg	Musmus IGHJ4*01	Musmus IGHD3-1*01
171-C03_Ms	Musmus IGHV11-2*02	tgtatgagata				.......tggtaa....				ctactggtacttcgatgtctgg	Musmus IGHJ1*01	Musmus IGHD2-1*01
172-E02_Ms	Musmus IGHV5-12-2*01	tgtgcaagaca				.......tggtaacta.				.ctggtttgcttactgg	Musmus IGHJ3*01	Musmus IGHD2-1*01
174-C01_Ms	Musmus IGHV1-9*01	tgtgcaaga				..tactatagg......				..tacgatgctatggactactgg	Musmus IGHJ4*01	Musmus IGHD2-14*01
155-A07_Ms	Musmus IGHV2-3*01	tgtgccaaacc		t		.....tatggtaactac		cccct		.ttactatgctatggactactgg	Musmus IGHJ4*01	Musmus IGHD2-1*01

155-B10_Ms	Musmus IGHV2-6-7*01	tgtgccag...				.......ctcgggct..	cc		.....tttgcttactgg	Musmus IGHJ3*01	Musmus IGHD3-1*01
155-D08_Ms	Musmus IGHV2-9-1*01	tgtgcc.....		ccccag		...........ggtagtagct..	ccct		....gtttgcttactgg	Musmus IGHJ3*01	Musmus IGHD1-1*01
156-E03_Ms	Musmus IGHV2-6*03	tgtgccag.		ct		......ctaccct...............	t		.....tatgctatggactactgg	Musmus IGHJ4*01	Musmus IGHD5-7*01
156-F03_Ms	Musmus IGHV5-9-1*01	tgtgcaaga		ct		.......tggtaactac	tt		.ttactatgctatggactactgg	Musmus IGHJ4*01	Musmus IGHD2-1*01
156-G08_Ms	Musmus IGHV5-4*02	tgtgcaaga..		ac		cctactacgg.......			..tactatgctatggactactgg	Musmus IGHJ4*01	Musmus IGHD2-10*01
156-G10_Ms	Musmus IGHV5-12-2*01	tgtgcaagaca				...actggg..	ggc		....ctatgctatggactactgg	Musmus IGHJ4*01	Musmus IGHD4-1*01
156-G12_Ms	Musmus IGHV3-2*02	tgtgcaag.		gggag		........aggtacgac	gtcct		.....tttgcttactgg	Musmus IGHJ3*01	Musmus IGHD2-14*01
156-H02_Ms	Musmus IGHV2-3*01	tgtgcca....				cctactatggtaact..	c		.ttactatgctatggactactgg	Musmus IGHJ4*01	Musmus IGHD2-10*01
156-H04_Ms	Musmus IGHV5-9-3*01	tgtgcaagac.				.........cggg....	gggggg		...ctggtacttcgatgtctgg	Musmus IGHJ1*01	Musmus IGHD3-1*01
156-H05_Ms	Musmus IGHV2-2*02	tgtgcgagaa.		gccctaa		.ctactataggta....			.ctggtttgcttactgg	Musmus IGHJ3*01	Musmus IGHD2-14*01
171-A02_Ms	Musmus IGHV5-9-4*01	tgtgcaa....		aaa		......atggtaactac	gtagga		........gctatggactactgg	Musmus IGHJ4*01	Musmus IGHD2-1*01
171-A10_Ms	Musmus IGHV2-5*01	tgtgcc.....		c		tctactatggttacg..	gga		attactatgctatggactactgg	Musmus IGHJ4*01	Musmus IGHD2-2*01
171-A11_Ms	Musmus IGHV6-6*01	tgtaccag.		aggggtc		..cattactacggc...	c		cctggtttgcttactgg	Musmus IGHJ3*01	Musmus IGHD1-2*01
171-A12_Ms	Musmus IGHV5-9-2*01	tgtgcaagaca		gag		....ttactacggtagtagc...	cc		....gtttgcttactgg	Musmus IGHJ3*01	Musmus IGHD1-1*01
171-D07_Ms	Musmus IGHV5-17*02	tgtgcaaga		aaggc		....ctataggtacga.	g		..tggtttgcttactgg	Musmus IGHJ3*01	Musmus IGHD2-14*01
172-E09_Ms	Musmus IGHV1-26*01	tgtgcaaga		cagc		tctactatggttacgac	gcgg		...ggtttgcttactgg	Musmus IGHJ3*01	Musmus IGHD2-2*01
172-F02_Ms	Musmus IGHV5-6*01	tgtgcaagaca		ggggc		...attactacggtagtagct..	ggg		......atgctatggactactgg	Musmus IGHJ4*01	Musmus IGHD1-1*01
172-F12_Ms	Musmus IGHV5-12-2*01	tgtgcaagac.		t		......atggttactac	cgc		..actggtacttcgatgtctgg	Musmus IGHJ1*01	Musmus IGHD2-3*01
172-G03_Ms	Musmus IGHV5-9-1*01	tgtgcaaga				.........cctatgc...........	ccc		attactatgctatggactactgg	Musmus IGHJ4*01	Musmus IGHD5-7*01
172-H05_Ms	Musmus IGHV1-18*01	tgtgcaaga		agggaggg		....gtatggtaactac	tt		.ctggtttgcttactgg	Musmus IGHJ3*01	Musmus IGHD2-10*02
174-A08_Ms	Musmus IGHV2-5-1*01	tgtgccaaaaa				....ctatgattacga.	agggg		...ggtttgcttactgg	Musmus IGHJ3*01	Musmus IGHD2-4*01
174-B08_Ms	Musmus IGHV5-6*01	tgtgc......		c		..tattactac...........	tc		....ctatgctatggactactgg	Musmus IGHJ4*01	Musmus IGHD1-1*01
155-A12_Ms	Musmus IGHV2-2*02	tgtgccagaaa	t	g		........gattac...	cg		.....tatgctatggactactgg	Musmus IGHJ4*01	Musmus IGHD2-4*01
155-B02_Ms	Musmus IGHV1-14*01	tgtgcaaga	tc	ggga		.....tactacggtagtagc...	cc		....gtttgcttactgg	Musmus IGHJ3*01	Musmus IGHD1-1*01
155-B11_Ms	Musmus IGHV6-6*02	tgtaccagg	c	ggg		..aactgggac	cctt		....ctatgctatggactactgg	Musmus IGHJ4*01	Musmus IGHD4-1*01
156-F09_Ms	Musmus IGHV2-6-5*01	tgtgccaaaca	t	tcc		...........gtgg.......			cctggtttgcttactgg	Musmus IGHJ3*01	Musmus IGHD1-1*02
156-G02_Ms	Musmus IGHV1S16*01	tgtacaa..	gg			........ggtaac...			..tactatgctatggactactgg	Musmus IGHJ4*01	Musmus IGHD2-1*01
156-G03_Ms	Musmus IGHV4-1*02	tgtgcaagacc	gg	gt		..tatgatggttac...	c		cctggtttgcttactgg	Musmus IGHJ3*01	Musmus IGHD2-3*01
156-G04_Ms	Musmus IGHV5-12-2*01	tgtgcaagaca	tg	ga		..........gggggtaactac	gg		.......tgcttactgg	Musmus IGHJ3*01	Musmus IGHD1-1*02
172-E07_Ms	Musmus IGHV14-4*02	tgtaatgca	tg	ggc		....ctatggtaactac	gtaaga		.....tttgcttactgg	Musmus IGHJ3*01	Musmus IGHD2-1*01
172-G02_Ms	Musmus IGHV2-9-1*01	tgtgccagaga	tc	gct		.......ctcgggc...			....ctttgactactgg	Musmus IGHJ2*01	Musmus IGHD3-1*01
172-G10_Ms	Musmus IGHV1-66*01	tgtgcaaga	tc	gggg		..........ggtagtagctac	cccc		......ttgactactgg	Musmus IGHJ2*01	Musmus IGHD1-1*01
174-B07_Ms	Musmus IGHV14-4*02	tgtaatgca	tg	ggc		....ctatggtaactac	gtaaga		.....tttgcttactgg	Musmus IGHJ3*01	Musmus IGHD2-1*01
155-C10_Ms	Musmus IGHV1-18*01	tgtgcaag.		ggg	gg	cctactatggtaac...	c		cctggtttgcttactgg	Musmus IGHJ3*01	Musmus IGHD2-10*01
171-B09_Ms	Musmus IGHV1-14*01	tgtgcaaga		c		.......ctaactacgtag....	aa	g	cctggtttgcttactgg	Musmus IGHJ3*01	Musmus IGHD1-3*01
171-D12_Ms	Musmus IGHV2-9-1*01	tgtgccagag.		tg		..........ggtggtaactac	gtacgt	t	attactatgctatggactactgg	Musmus IGHJ4*01	Musmus IGHD1-1*02
172-F03_Ms	Musmus IGHV1-54*01	tgtgcaaga				...agaagggt......	ct	g	cctggtttgcttactgg	Musmus IGHJ3*01	Musmus IGHD2-10*02
156-E06_Ms	Musmus IGHV5-12-2*01	tgtgcaagac.				.taactgggac		gt	actactttgactactgg	Musmus IGHJ2*01	Musmus IGHD4-1*01
172-F09_Ms	Musmus IGHV5-6-4*01	tgtacaagaga	t			.ggcactgggcc................	c	t	actactttgactactgg	Musmus IGHJ2*01	Musmus IGHD5-7*01

CD138- Splenic B-1a Cells

Input	V name	3'V-REGION	P	N1	P	D-REGION	P	N2	P	5'J-REGION	J name	D name
173-G07	Musmus IGHV1-15*01	tgtacaaga	t	tgagg		........gattacgac		gaggg	gt	actactttgactactgg	Musmus IGHJ2*01	Musmus IGHD2-4*01
171-G02	Musmus IGHV1-18*01	tgtgca...				....gtatgg.......				..tactttgactactgg	Musmus IGHJ2*01	Musmus IGHD2-10*02
172-D08	Musmus IGHV1-26*01	tgtgcaaga				.....................cagaacc		ca	t	actactttgactactgg	Musmus IGHJ2*01	Musmus IGHD5-7*01
173-H02	Musmus IGHV1-39*01	tgtgcaaga				.....tatggta.....				...actatgctatggactactgg	Musmus IGHJ4*01	Musmus IGHD2-1*01
172-A01	Musmus IGHV1-4*01	tgtgcaaga				..........tgggggttacta.			a	cctggtttgcttactgg	Musmus IGHJ3*01	Musmus IGHD1-1*02
171-H03	Musmus IGHV14-3*02	tgtgctag.		tcc		cctacttaggta.....			tc	actactttgactactgg	Musmus IGHJ2*01	Musmus IGHD2-10*01
172-B10	Musmus IGHV1-55*01	tgtgcaaga				..tattactacggtagtagc...			ggggg	....ctatgctatggactactgg	Musmus IGHJ4*01	Musmus IGHD1-1*01
172-E01	Musmus IGHV1-69*02	tgtacaaga				........................aacc			cc	..........atggactactgg	Musmus IGHJ4*01	Musmus IGHD5-7*01
173-H05	Musmus IGHV1-7*01	tgtgcaaga			t	..........cccct.............				.tattggtacttcgatgtctgg	Musmus IGHJ1*01	Musmus IGHD5-7*01
172-D01	Musmus IGHV1-7*01	tgtgcaaga	tc	a		......actacggtagtagct..		tc		.......tgactactgg	Musmus IGHJ2*01	Musmus IGHD1-1*01
171-F03	Musmus IGHV1-9*01	tgtgcaaga				..tactataggt.....				acgactttgactactgg	Musmus IGHJ2*01	Musmus IGHD2-14*01
172-D10	Musmus IGHV2-9-1*01	tgtgccag...		t		.........gttact..			ggt	..........tactgg	Musmus IGHJ2*01	Musmus IGHD2-3*01
172-A02	Musmus IGHV2-9-1*01	tgtgccag...		tc	aa	tttattactacggtagtagct..		tccga		............ggactactgg	Musmus IGHJ4*01	Musmus IGHD1-1*01
172-C02	Musmus IGHV3-1*02	tgtgcaaga				.......ctacgg..........				.tactggtacttcgatgtctgg	Musmus IGHJ1*01	Musmus IGHD1-1*01
171-G07	Musmus IGHV3-2*02	tgtgcaaga		ggag	a	ttcattactacggct..		gg		actactttgactactgg	Musmus IGHJ2*01	Musmus IGHD1-2*01
172-A05	Musmus IGHV3-2*02	tgtgcaaga		ggccg		....ctataggtac...			ctc	.....tttgcttactgg	Musmus IGHJ3*01	Musmus IGHD2-14*01
172-D07	Musmus IGHV3-6*02	tgtgcaagaga	t	gtc		..cattactacggctac		ctc	t	attactatgctatggactactgg	Musmus IGHJ4*01	Musmus IGHD1-2*01
171-H10	Musmus IGHV3-8*02	tgtgcaagata	ta	gaggg		.....tactacggtagtag....				.ctactttgactactgg	Musmus IGHJ2*01	Musmus IGHD1-1*01
172-C10	Musmus IGHV5-17*02	tgtgcaaga				.....tatggta.....				...actacgctatggactactgg	Musmus IGHJ4*01	Musmus IGHD2-1*01
172-C12	Musmus IGHV5-6*01	tgtgcaagaca	tg	gg		...actatgatta....				.........ctctggactactgg	Musmus IGHJ4*01	Musmus IGHD2-4*01
172-D04	Musmus IGHV5-6-2*01	tgtgcaagaca				.......ctacggtagta.....				.....ggtacttcgatgtctgg	Musmus IGHJ1*01	Musmus IGHD1-1*01
172-A09	Musmus IGHV5-6-2*01	tgtgcaa....		a		.ctactatggtaactac			g	...ggtttgcttactgg	Musmus IGHJ3*01	Musmus IGHD2-1*01
171-G11	Musmus IGHV6-6*01	tgtaccagg			c	gggac............			g	cctggtttgcttactgg	Musmus IGHJ3*01	Musmus IGHD3-3*01
172-C03	Musmus IGHV6-6*02	tgtacc...				..........ggtgg.......				............ggactactgg	Musmus IGHJ4*01	Musmus IGHD1-1*02
124-A05_Ms	Musmus IGHV10-1*02	tgtgtgagaca		gg		....cagctcgg.....			t	actactttgactactgg	Musmus IGHJ2*01	Musmus IGHD3-1*01
120-F05_Ms	Musmus IGHV10-1*02	tgtgtgagaca			tg	....ctggg..				....tggtacttcgatgtctgg	Musmus IGHJ1*01	Musmus IGHD4-1*01
125-E01_Ms	Musmus IGHV10S3*01	tgtgtgagaga	tc	a		.ttattactacggtagtagctac		a		cctggtttgcttactgg	Musmus IGHJ3*01	Musmus IGHD1-1*01
120-G01_Ms	Musmus IGHV11-2*02	tgtatgagata				............tagtag....				ctactggtacttcgatgtctgg	Musmus IGHJ1*01	Musmus IGHD1-1*01
120-F07_Ms	Musmus IGHV1-14*01	tgtgcaaga		ggggg		..........gggc...				........gctatggactactgg	Musmus IGHJ4*01	Musmus IGHD3-1*01
121-B03_Ms	Musmus IGHV1-14*01	tgtgcaaga		gggg		......actacggtag.......		c	g	cctggtttgcttactgg	Musmus IGHJ3*01	Musmus IGHD1-1*01
124-C10_Ms	Musmus IGHV1-14*01	tgtgcaaga		gaggaa		..tatgatggttact..				actactttgactactgg	Musmus IGHJ2*01	Musmus IGHD2-3*01
121-B06_Ms	Musmus IGHV1-26*01	tgtgcaaga	tc	agggg		..tattactacgg..........		cccg		attactatgctatggactactgg	Musmus IGHJ4*01	Musmus IGHD1-1*01
125-E03_Ms	Musmus IGHV1-39*01	tgtgcaaga		gtc		...........aggggtaact..		tccc		....ctttgactactgg	Musmus IGHJ2*01	Musmus IGHD1-3*01
120-G07_Ms	Musmus IGHV1-4*01	tgtgcaaga		g		..........gggc...				....gtttgcttactgg	Musmus IGHJ3*01	Musmus IGHD3-1*01
125-H11_Ms	Musmus IGHV1-4*01	tgtgcaaga		gt		.......ctacggtagtagctac		tgg		..tactatgctatggactactgg	Musmus IGHJ4*01	Musmus IGHD1-1*01
125-G06_Ms	Musmus IGHV1-4*01	tgtgcaag.		gggac		............tggcggctac			gg	cctcgtttgcttactgg	Musmus IGHJ3*01	Musmus IGHD1-1*02
120-E07_Ms	Musmus IGHV14-2*01	tgtgct...		g		......atggttacg..		c	gg	cctggtttgcttactgg	Musmus IGHJ3*01	Musmus IGHD2-2*01

124-C12_Ms	Musmus IGHV14-3*02	tgtgctgga				...attactacggtagta.....	a		actactttgactactgg	Musmus IGHJ2*01	Musmus IGHD1-1*01
122-F08_Ms	Musmus IGHV1-5*01	tgtac....		tgttggggcgg		...atgatgg.......			....ctttgactactgg	Musmus IGHJ2*01	Musmus IGHD2-3*01
124-B10_Ms	Musmus IGHV1-54*01	tgtgcaaga				.....tact.............			atgactatgctatggactactgg	Musmus IGHJ4*01	Musmus IGHD1-1*01
121-A03_Ms	Musmus IGHV1-54*01	tgtgcaaga		agaggcc		cctactataggtacgac	ggg		........gactactgg	Musmus IGHJ2*01	Musmus IGHD2-14*01
121-C06_Ms	Musmus IGHV1-63*02	tgtgccaga				...actgggac	ctt		....ctatgctatggactactgg	Musmus IGHJ4*01	Musmus IGHD4-1*01
121-D03_Ms	Musmus IGHV1-7*01	tgtgcaaga				...........gggggt......			.....tttgcttactgg	Musmus IGHJ3*01	Musmus IGHD1-1*02
120-F12_Ms	Musmus IGHV1-80*01	tgtgcaaga		a		..gaccactacggcagt			actactttgactactgg	Musmus IGHJ2*01	Musmus IGHD3-3*01
120-G10_Ms	Musmus IGHV1-80*01	tgtgcaaga		gaggagggg		........aggtacgac	at		.ctggtttgcttactgg	Musmus IGHJ3*01	Musmus IGHD2-14*01
120-G08_Ms	Musmus IGHV1-9*01	tgtgcaa..		acct		.....ctatggtaactac	gagg		...actttgactactgg	Musmus IGHJ2*01	Musmus IGHD2-1*01
124-A02_Ms	Musmus IGHV1S16*01	tgtacaa..				gagta.....			..tactatgctatggactactgg	Musmus IGHJ4*01	Musmus IGHD5-1*01
125-H03_Ms	Musmus IGHV1S16*01	tgtacaata				tctactatgattacgac	g		..tggtttgcttactgg	Musmus IGHJ3*01	Musmus IGHD2-4*01
124-B07_Ms	Musmus IGHV2-3*01	tgtgcca....		tc		..tattactacggtagtagc...	ct		.ttactatgctatggactactgg	Musmus IGHJ4*01	Musmus IGHD1-1*01
125-F05_Ms	Musmus IGHV2-3*01	tgtgcca....		ccc		...atgatggtt.....			acttctttgactactgg	Musmus IGHJ2*01	Musmus IGHD2-3*01
121-C09_Ms	Musmus IGHV2-3*01	tgtgccaaa..		g		......actacggtagtagct..	gggg	at	attactatgctatggactactgg	Musmus IGHJ4*01	Musmus IGHD1-1*01
121-D09_Ms	Musmus IGHV2-5*01	tgtgccaaaaa			aga	tctactatgattacgac	g		attactatgctatggactactgg	Musmus IGHJ4*01	Musmus IGHD2-4*01
120-F06_Ms	Musmus IGHV2-6*02	tgtgccaga..		tg		....ttactacgg..........			....ctacgctatggactactgg	Musmus IGHJ4*01	Musmus IGHD1-1*01
120-H03_Ms	Musmus IGHV2-6-1*01	tgtgccaga..				...................gccac....			....ctttgactactgg	Musmus IGHJ2*01	Musmus IGHD5-7*01
121-B02_Ms	Musmus IGHV2-6-4*01	tgtgccaga..		ggcc		.......ctacggct..	gatgg		.ttactatgctatggactactgg	Musmus IGHJ4*01	Musmus IGHD1-2*01
124-D04_Ms	Musmus IGHV2-6-7*01	tgtgccagag.		tgg		.........ggtacga.	ggg		.ctggtttgcttactgg	Musmus IGHJ3*01	Musmus IGHD2-14*01
120-E11_Ms	Musmus IGHV2-9-1*01	tgtgcc.....				.....tatggttac...			.....tttgcttactgg	Musmus IGHJ3*01	Musmus IGHD2-2*01
121-B09_Ms	Musmus IGHV2-9-1*01	tgtgccaga..				.....tactacggtagtagc...			..tactatgctatggactactgg	Musmus IGHJ4*01	Musmus IGHD1-1*01
125-E12_Ms	Musmus IGHV2-9-1*01	tgtgccagaga				....ttactacggtagtag....			ctactggtacttcgatgtctgg	Musmus IGHJ1*01	Musmus IGHD1-1*01
120-H10_Ms	Musmus IGHV2-9-1*01	tgtgccagag.		gggg		....ttactacggtagtagc...			..tacgatgctatggactactgg	Musmus IGHJ4*01	Musmus IGHD1-1*01
121-C01_Ms	Musmus IGHV2-9-1*01	tgtgccagag.		ccg		.....gatgggtcct..			....ctatgctatggactactgg	Musmus IGHJ4*01	Musmus IGHD2-3*01
124-A06_Ms	Musmus IGHV2-9-1*01	tgtgccag...		g		...attacgacgtgggt......			.ttactatgctatggactactgg	Musmus IGHJ4*01	Musmus IGHD1-1*02
124-D01_Ms	Musmus IGHV2-9-1*01	tgtgccagaga	t	atgagg		.....tataggtacgac	gcctt		.ttactatgctatggactactgg	Musmus IGHJ4*01	Musmus IGHD2-14*01
124-D05_Ms	Musmus IGHV2-9-1*01	tgtgccagaga	t	ggaggga	a	tctactatggttacgac	agggga		...actttgactactgg	Musmus IGHJ2*01	Musmus IGHD2-2*01
124-A09_Ms	Musmus IGHV3-1*02	tgtgcaaga		agaagaatg		.....tactacggtagtag....			...ctggtacttcgatgtctgg	Musmus IGHJ1*01	Musmus IGHD1-1*01
121-D12_Ms	Musmus IGHV3-2*02	tgtgcaag.		c		......actacggtagtag....	agg		..actggtacttcgatgtctgg	Musmus IGHJ1*01	Musmus IGHD1-1*01
125-G03_Ms	Musmus IGHV3-2*02	tgtgcaag.		ttt		.......tggtaac...	cc		....gtttgcttactgg	Musmus IGHJ3*01	Musmus IGHD2-1*01
124-D03_Ms	Musmus IGHV3-2*02	tgtgcaaga			tc	..........aaggggta.....			actactttgactactgg	Musmus IGHJ2*01	Musmus IGHD1-3*01
125-F09_Ms	Musmus IGHV3-2*02	tgtgcaaga			g	ctaactgggac		t	actactttgactactgg	Musmus IGHJ2*01	Musmus IGHD4-1*01
120-G12_Ms	Musmus IGHV3-6*02	tgtgcaagag.		g		........gattacgac	gaaga		actactttgactactgg	Musmus IGHJ2*01	Musmus IGHD2-4*01
125-E08_Ms	Musmus IGHV3-6*02	tgtgcaagag.		ccccaa		......ataggtacgac	aaggg		....ctatgctatggactactgg	Musmus IGHJ4*01	Musmus IGHD2-14*01
124-B05_Ms	Musmus IGHV3-6*02	tgtgcaag...		ca		......actacggtagtagct..	t	tag	ctactggtacttcgatgtctgg	Musmus IGHJ1*01	Musmus IGHD1-1*01
124-B12_Ms	Musmus IGHV3-6*02	tg.........		c		..tattactacggtagtagctac		t	attactatgctatggactactgg	Musmus IGHJ4*01	Musmus IGHD1-1*01
120-E05_Ms	Musmus IGHV3-8*02	tgtgcaaga..		ggga		......atggtaactac	gtgcc		.......tgcttactgg	Musmus IGHJ3*01	Musmus IGHD2-1*01
120-E04_Ms	Musmus IGHV4-1*02	tgtgcaagac.		ga		........ggtaac...	cctc		actactttgactactgg	Musmus IGHJ2*01	Musmus IGHD2-1*01
124-B11_Ms	Musmus IGHV5-12-1*01	tgtgcaagaca		acgg		........ggtaacta.			.ctggtttgcttactgg	Musmus IGHJ3*01	Musmus IGHD2-1*01
124-B04_Ms	Musmus IGHV5-17*02	tgtgcaaga			tc	....ctggg..			actactttgactactgg	Musmus IGHJ2*01	Musmus IGHD4-1*01
120-E03_Ms	Musmus IGHV5-2*01	tgtgcaaga..				........cagggta.			...actacgctatggactactgg	Musmus IGHJ4*01	Musmus IGHD3-2*02
121-A11_Ms	Musmus IGHV5-2*01	tgtgcaaga..		c		...atgatgg.......	ccccc		....ctatgctatggactactgg	Musmus IGHJ4*01	Musmus IGHD2-3*01
120-G11_Ms	Musmus IGHV5-4*02	tgtgcaagaga	t			....caggggggg....	tca		..tactatgctatggactactgg	Musmus IGHJ4*01	Musmus IGHD3-1*01
121-B01_Ms	Musmus IGHV5-4*02	tgtgcaagag.	cgg	gggacggcta.......		ctc	gg		cctggtttgcttacggg	Musmus IGHJ3*01	Musmus IGHD3-3*01
124-D10_Ms	Musmus IGHV5-6*01	tgtgcaagaca				....ttactacggtagtag....			....ctatgctatggactactgg	Musmus IGHJ4*01	Musmus IGHD1-1*01
120-G04_Ms	Musmus IGHV5-6-2*01	tgtgcaagaca	tg	ggggct		...attactacggctac	cca		.....tttgactactgg	Musmus IGHJ2*01	Musmus IGHD1-2*01
121-A09_Ms	Musmus IGHV5-6-3*01	tgtgcaagaga				.............aggag....			cctggtttgcttactgg	Musmus IGHJ3*01	Musmus IGHD1-1*01
124-D02_Ms	Musmus IGHV5-6-3*01	tgtgcaagaga				.ctactatggtaac...	ctc		.....tttgcttactgg	Musmus IGHJ3*01	Musmus IGHD2-1*01
121-C08_Ms	Musmus IGHV5-6-4*01	tgtacaagaga		g		.....tatgattac...	c		actactttgactactgg	Musmus IGHJ2*01	Musmus IGHD2-4*01
121-D08_Ms	Musmus IGHV5-9-3*01	tgtgcaagaca				....tgatggt......			.....tatgctatggactactgg	Musmus IGHJ4*01	Musmus IGHD2-3*01
120-F11_Ms	Musmus IGHV5-9-4*01	tgtggaaggga	t	gggaa		.ctacgataggtacg..	t		....ctatgcgatggactacggg	Musmus IGHJ4*01	Musmus IGHD2-14*01
124-B09_Ms	Musmus IGHV5S12*01	tgtgcaaga		gg		....ctatgattac...			........gactactgg	Musmus IGHJ2*01	Musmus IGHD2-4*01
121-B07_Ms	Musmus IGHV6-6*01	tgtaccagg				.....tatggtaact..			actactttgactactgg	Musmus IGHJ2*01	Musmus IGHD2-1*01
125-F07_Ms	Musmus IGHV7-1*02	tgtgcaagag.....				.........gttac............			..tactatgctatggactactgg	Musmus IGHJ4*01	Musmus IGHD2-12*01
121-B12_Ms	Musmus IGHV7-3*02	tgtgcaagagat.				...........ggta........			actactttgactactgg	Musmus IGHJ2*01	Musmus IGHD1-1*01
